# The NuRD Complex in Neurodevelopment and Disease: A Case of Sliding Doors

**DOI:** 10.3390/cells12081179

**Published:** 2023-04-18

**Authors:** Paraskevi Boulasiki, Xiao Wei Tan, Matteo Spinelli, Antonella Riccio

**Affiliations:** 1 UCL Laboratory for Molecular Cell Biology, University College London, London WC1E 6BT, UK; 2 Neuroscience Department, Catholic University of the Sacred Heart, 00168 Rome, Italy

**Keywords:** neuroepigenetics, chromatin remodelling, NuRD, CHD4, neurodevelopment, neurodegeneration

## Abstract

The Nucleosome Remodelling and Deacetylase (NuRD) complex represents one of the major chromatin remodelling complexes in mammalian cells, uniquely coupling the ability to “open” the chromatin by inducing nucleosome sliding with histone deacetylase activity. At the core of the NuRD complex are a family of ATPases named CHDs that utilise the energy produced by the hydrolysis of the ATP to induce chromatin structural changes. Recent studies have highlighted the prominent role played by the NuRD in regulating gene expression during brain development and in maintaining neuronal circuitry in the adult cerebellum. Importantly, components of the NuRD complex have been found to carry mutations that profoundly affect neurological and cognitive development in humans. Here, we discuss recent literature concerning the molecular structure of NuRD complexes and how the subunit composition and numerous permutations greatly determine their functions in the nervous system. We will also discuss the role of the CHD family members in an array of neurodevelopmental disorders. Special emphasis will be given to the mechanisms that regulate the NuRD complex composition and assembly in the cortex and how subtle mutations may result in profound defects of brain development and the adult nervous system.

## 1. Introduction

In all cells, the DNA is compacted in a higher order structure known as nucleosomes, comprising octamers of histone H2A, H2B, H3 and H4 dimers wrapped within a stretch of 147 DNA base pairs [1]. The state of chromatin and the “tightness” of the wrapping is instrumental in defining whether the DNA and the genes comprised within will be in an activated or repressed state. Euchromatin, which is the chromatin containing actively transcribed genes in a any given cell type, is usually characterised by a lower nucleosome density. In fact, nucleosome density usually correlates with accessibility to transcription and epigenetic factors. For example, promoters of transcriptionally active genes are notoriously devoid of nucleosomes [2,3]. Chromatin density, however, is for the most part highly dynamic, and nucleosomes are repositioned and remodelled in response to intracellular and extracellular stimuli [4,5].

The Nucleosome Remodelling and Deacetylase complex (NuRD) comprises a family of enzymes named chromodomain/helicase/DNA-binding (CHD) proteins, which are ATPases that use the energy released by the hydrolysis of ATP to induce nucleosome sliding and modify chromatin [6,7]. Although NuRD was originally described as mostly a transcriptional repressor complex, it is now recognised that it may modulate gene expression in multiple ways, acting as both a repressor and an activator [8,9]. CHDs are at the core of the NuRD complex; however, they can also act in the context of other chromatin-binding proteins. For instance, CHD4 can interact with ADNP and HP1γ to form the ChAHP complex [10].

A fundamental feature of chromatin remodelling complexes is that each component has several paralogs, implying that numerous combinations of the core subunits are possible. For example, the Switch/Sucrose Non-Fermenting chromatin remodelling complex SWI/SNF undergoes a paralog switching during neuronal development [11]. In mammals, the ATPases of the SWI/SNF complex are encoded by two paralog genes, Brm and Brg1, that undergo subunit switching during cell differentiation [12]. Additional subunits such as BAF53 are also exchanged when neuronal progenitors exit the cell cycle to achieve terminal differentiation [11,13]. So far, subunit switching has been mostly linked to the relative expression of paralogs that would account for their inclusion within the complex. However, it is now recognised that in many instances, paralogs may be expressed at similar levels and yet they would be included in chromatin remodelling complexes in a very specific manner [14,15]. Here, we will review recent literature indicating that posttranslational modifications of chromatin remodelling complexes’ subunits are essential in determining their assembly on the chromatin. We will focus on the NuRD complex due to its pivotal role in regulating the transcriptional programme during neurodevelopment and in specific neurological disorders.

## 2. The Plot

### 2.1. Setting the Scene: The NuRD Complex

**Figure 1:**
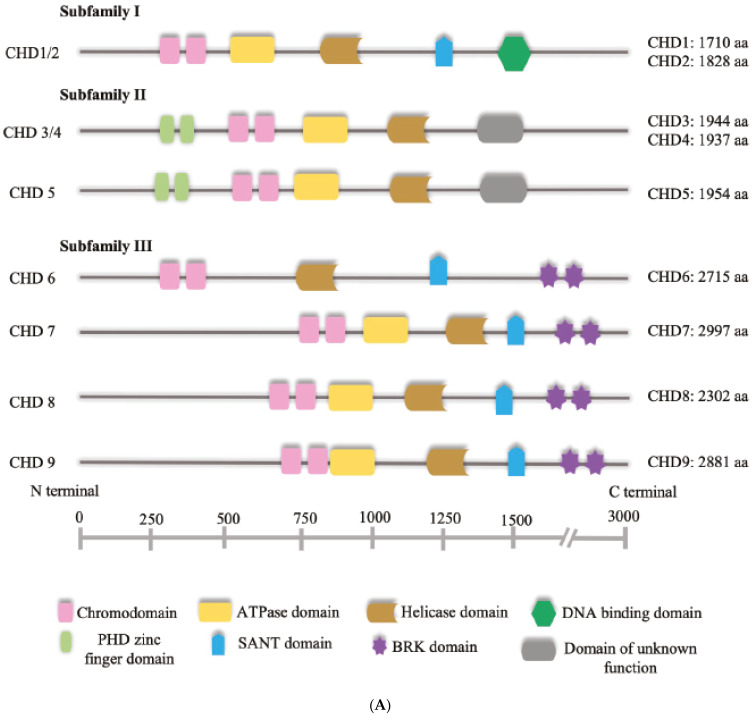

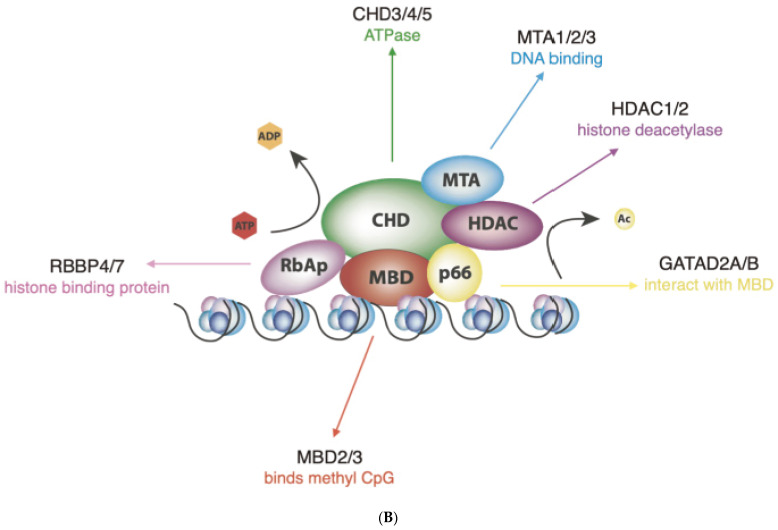
(**A**) Schematic representation of human CHD proteins subfamilies, showing the approximate size and all known or predicted domains. (**B**) The NuRD complex. The primary components of the NuRD complex include the ATP-dependent remodelling enzymes CHD3/4/5, the histone deacetylases HDAC1/2, the scaffold proteins MTA1/2/3, the histone chaperones RBBP4/7, the CpG-binding proteins MBD2/3, p66α and/or p66β.

The cornerstone of the NuRD complex is the chromodomain helicase DNA-binding protein, or CHD. CHDs are ATPases that utilise the energy produced from the hydrolysis of ATP to induce nucleosome sliding [16,17,18]. They are the central subunits of the NuRD complex and are divided into three subfamilies based on their structure [19] (Figure 1A). Family 1 comprises CHD1 and CHD2; Family 2 consists of CHD3, CHD4 and CHD5 and are the CHDs found to be included in NuRD; and family three includes CHD6, CHD7, CHD8 and CHD9. While the catalytic domain is conserved across families, they diverge at the DNA-binding domain, and may express additional elements such as the helical HMG-like domain [19] (Figure 1A). It has been shown that in some cases, CHDs may function in the absence of interaction with the other NuRD subunits, raising the question of whether the assembly into complexes is necessary for the nucleosome sliding activity. CHD4 for example, is capable of inducing nucleosome sliding in vitro and it is not required for the assembly of stable NuRD complexes [20,21]. Moreover, in cerebellar granule cells, CHD4 regulates chromatin accessibility by regulating chromatin loop formation with a mechanism that requires the recruitment of CTCF/cohesin complex to enhancers and it is likely to be independent of other subunits of the NuRD complex [22]. It should also be noted that although quite conserved structurally, CHDs may display different DNA-binding activity depending on both the presence of specific domains and the combination of subunit paralogs with which they are assembled. For example, RBBP4 and RBBP7 are histone-binding proteins of the NuRD complex which can also interact with other transcriptional regulators, potentially contributing to determine the genome binding sites of NuRD and thus CHD [15]. 

NuRD complexes uniquely couple ATP-dependent chromatin remodelling with histone deacetylase activity, by recruiting the histone deacetylases (HDAC) 1 or HDAC2 (Figure 1B) [18]. HDAC 1 and 2 bind extensively to the mammalian genome and are found in association with several transcription factors and chromatin-binding complexes, such as Sin3, CoREST and the Polycomb repressive complex [23]. MBD2 and MBD3 are DNA-binding proteins that possess different affinities for methylated DNA and are mutually exclusive within the NuRD complex [24]. Additional structural proteins include MTA1, MTA2 and MTA3 that act as the scaffold around which the NuRD complexes are assembled [25]. Given that NuRDs depleted of CHD are stable, bind chromatin and show deacetylase activity [20], it is possible that NuRD composition may have an impact on their function that goes beyond determining the chromatin-binding sites and may extend to whether they induce nucleosome sliding or only histone epigenetic modifications. Thus, one of the most important challenges of future research will be to determine the exact subunit composition of NuRD complexes on specific genomic sites, considering not only the paralogs included but also the potential absence of ATPases altogether, as it will have a profound impact on how NuRD regulates gene expression.

### 2.2. Finding the Location: NuRD Complex in Developing and Adult Neurons

**Figure 2:**
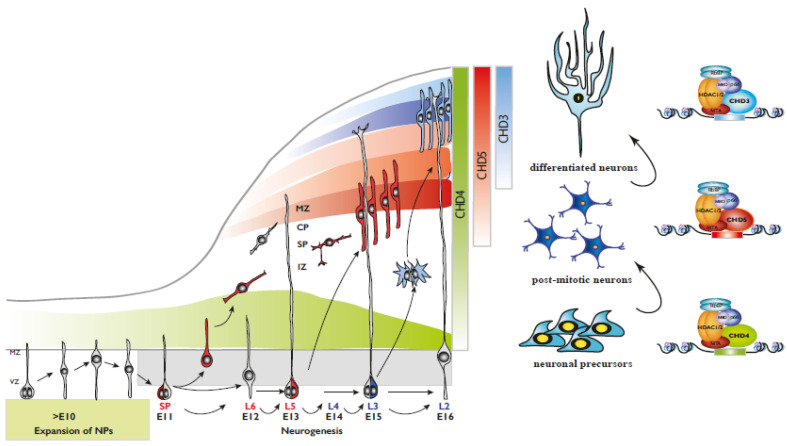
(**Left**) Representation of mammalian corticogenesis. Arrows indicate lineage relationships. Neuroepithelial cells undergo symmetric cell division to produce an initial pool of cortical progenitors that later transform into ventricular radial glial cells (vRGCs). vRGCs divide asymmetrically and generate another vRGC and a nascent projection neuron. The neuron migrates radially from the ventricular zone (VZ) along the basal process of an RGC into the cortical plate (CP). The earliest born neurons migrate to form the preplate and later migrating neurons split the preplate into the marginal zone (MZ) and the subplate (SP). IZ, intermediate zone; SP, subplate; CP, cortical plate; MZ, marginal zone. (**Right**) CHD paralog switching during corticogenesis.

In mammalians, the development of the nervous system is a complex task requiring multiple steps precisely coordinated over a long time that in humans can span years [26]. The cortex of higher mammals is formed by an intricate process that starts with the proliferation of self-renewing neuronal progenitors within the ventricular zone of the developing brain (Figure 2). Over a few weeks, which would be months in the case of the human brain, neural progenitors exit the cell cycle and start migrating outwards, thus populating the deep layers of the cortex first, in an inside–out process where late-born neurons must cross the deeper layers to reach their final position in a more superficial layer. An in-depth description of mammalian cortical development is beyond the scope of this review, but for recent comprehensive literature on the topic, please see [26,27,28,29] (Figure 2).

The NuRD complex has been shown to participate in all phases of brain development, from neuronal progenitor expansion, neuronal differentiation and migration to the establishment and maintenance of neuronal connectivity [14,30]. During the early phases of cortical development, neuronal progenitors located in the ventricular zone of the brain undergo asymmetric division to generate both neuronal precursors that will differentiate, and other progenitors that will replenish the proliferative pool. Conditional deletion of CHD4 in the murine brain results in a dramatic decrease in neuronal progenitors and a decrease in the thickness of the cortex [8,14]. Interestingly, mutations and deletions of CHD4 in humans have the opposite effect with patients showing, among other morphological defects, a remarkable overgrowth of the brain [31]. A potential explanation may reside in the potential difference in transcriptional factors that NuRD interacts with in rodents or humans. In mice, for example, the NuRD complex is known to interact and cooperate with the repressive Polycomb complex, to control the switch from neurogenesis to gliogenesis by inducing the recruitment of Polycomb to the promoter of genes responsible for neurogenesis, such as Neurogenin1 [32]. However, little is known regarding the nature of the transcription factors interacting with NuRD or the genes specifically regulated in the human brain. 

A second possible explanation is that the core subunits of the NuRD complex, the CHDs, may interact with distinct paralogs giving rise to complexes with different affinities for transcription factors or chromatin in rodents or humans. For example, the MTA1/2/3 paralogs act as scaffolding subunits that recognise several histone modifications. However, they are differentially included within NuRD complexes during cortical development with MTA1 becoming less abundant at later stages and MTA3 almost undetectable throughout the entire corticogenesis [14]. Given that MTAs also regulate the recruitment of other subunits to the NuRD, such as RBBP7 and HDAC1, it is conceivable that they may interact with gene promoters and chromatin loci in a species-specific manner.

It should also be noted that a further source of specificity may derive from the fact that CHDs often act independently of the NuRD complex. The ability of CHD4 to suppress astroglial differentiation during cortical development depends, at least in part, on its interaction with the Polycomb Repressor Complex PRC2 [33]. In mouse ES cells, a complex formed by CHD4 interaction with the transcription factor ADNP and the chromatin-associated protein HP1β/γ drives correct cell lineage differentiation [34]. Interestingly, in this case, the complex named ChAHP does not include the HP1 paralog α, perhaps suggesting that in other species, a subunit switch of this CHD4-containing complex may account for the regulation of distinct neuronal genes. 

**Figure 3:**
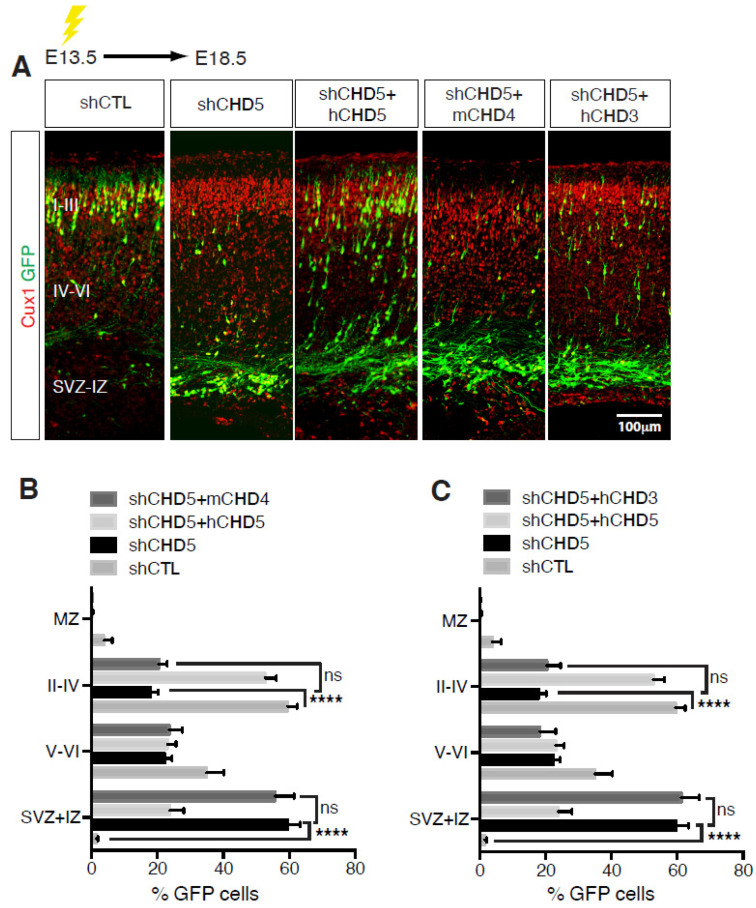

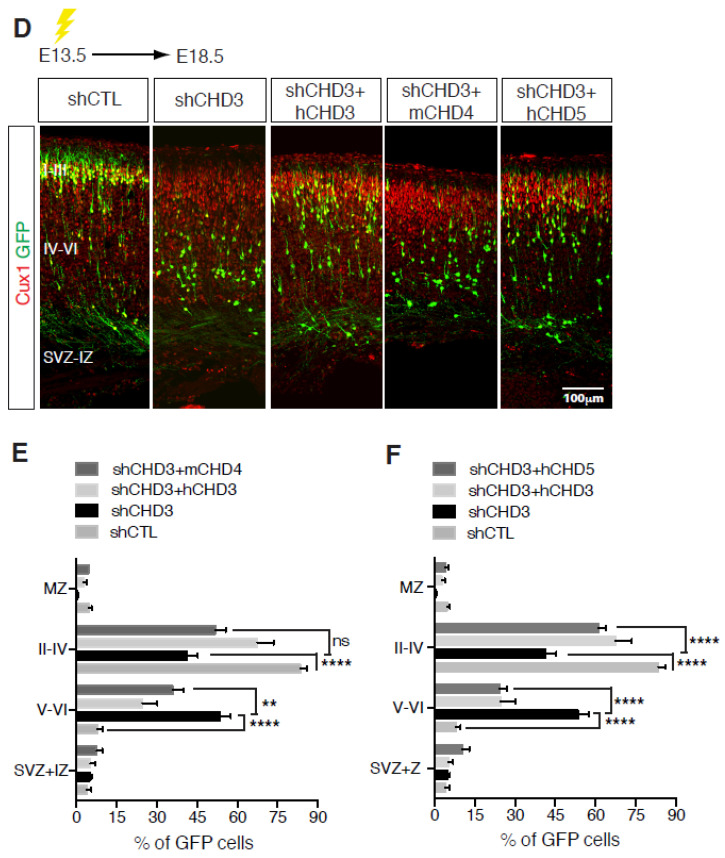
Non-redundant functions of CHDs during cortical development. (**A**,**D**) E13.5 embryos were electroporated with the indicated vectors and immunolabelled with GFP (green) and Cux1 (red) antibodies at E18.5. Scale bar, 100 μm. (**B**,**C**,**E**,**F**) Distribution of cells electroporated with the indicated vectors at E13.5 and analysed at E18.5. 8–13 embryos were analysed per condition; n = 3. All data are presented as mean ± SEM. ** *p* < 0.01, and **** *p* < 0.0001 (ns, not significant) by two-way ANOVA with Tukey’s multiple comparisons test. Data published in [14].

Once neuronal progenitors have exited the cell cycle, they start migrating radially toward the surface of the nascent cortex to populate the various cortical layers (Figure 2). Cortical migration is a unique process in that the first wave of neurons generated populate the deeper cortical layers, whereas neurons generated at later times must travel through the inner layers to reach the surface of the cortex [26,29] (Figure 2). CHD paralog switching is a powerful regulator of neuronal differentiation and radial migration. CHD3, CHD4 and CHD5 play distinct roles during mouse cortical development [14]. Our laboratory showed that the three paralogs display a distinct pattern of expression during mouse cortical development, with CHD4 expression much higher during the early stages of development and declining at later times [14]. Conversely, CHD3 and CHD5 are detected at relatively low levels at embryonic day E12.5, later becoming the prevalent paralogs as cortical development proceeds. Deletion of CHD4 reduces NPC proliferation, resulting in defects of cortical lamination and microcephaly. Interestingly, it specifically affects intermediate progenitor cells (IPCs), a class of basal progenitors that in mice are important neurogenic cells and may be responsible for the evolutionarily driven expansion of the mammalian cortex. Conversely, CHD3 and CHD5, although concomitantly expressed in the developing cortex, differentially regulate layer specification. Neuronal differentiation, migration and laminar specification are highly coordinated and co-regulated processes. Loss of CHD5 induced a delay of early neural migration with many neurons forming an ectopic layer and never reaching the outer layer of the cortex. In contrast, CHD3 controls late radial migration and layer specification. Thus, despite overlapping expression patterns, CHD3 and CHD5 have very distinct roles during cortical development [14]. Importantly, CHD3, CHD4 and CHD5 have mostly non-redundant functions in the brain given that the cortical abnormalities observed in the absence of any of them cannot be rescued by the overexpression of alternative paralogs (Figure 3). 

A potential limitation of this study, as well as for most studies analysing the role of the NuRD complex in mammalian cells, is that it is difficult to distinguish between the role of CHDs in the context of the NuRD complex from potential functions that may be due to the direct interaction of CHD with transcription factors. In a model of neurogenesis in vitro, CHD5 was found to contribute to the repression of Polycomb regulated genes [35]. Inhibition of CHD5 resulted in the dysregulation of neuronal genes and stunted differentiation of ES cells into neurons. Thus, although we found that CHDs co-immunoprecipitated with most NuRD subunits in the developing cortex [14], further analyses will be needed to unequivocally prove that this is the principal mechanism of action.

CHD4 has also been extensively studied in the adult cerebellum where it plays a critical role in mediating the expression of activity-induced genes [36]. CHD4 was found to occupy the promoter of transcriptionally active genes in adult rodent cerebellar granule cells, where it affected their inactivation following short bursts of depolarisation. Interestingly, cerebellar granule neurons lacking CHD4 showed a marked decrease in the histone isoform H2A.z, a histone variant associated with transcription and highly enriched at promoters [36]. An even wider role of CHD4 was revealed by the widespread increase in chromatin accessibility observed in the postnatal cerebellum of transgenic mice lacking CHD4. In addition to the previously observed binding to promoters, CHD4 was also found to be highly enriched at enhancers where it inhibits chromatin accessibility and the transcription of enhancer RNAs [22]. Importantly, CHD4 plays a key role in regulating the binding of chromatin to the cohesin complex, thereby mediating higher order genomic looping and nuclear architecture [22]. As mentioned above, a limitation of these studies is that they do not provide definitive evidence that in cerebellar granule cells, CHD4 acts in the context of the NuRD complex. This is especially important given that CHD4 ability to regulate chromatin looping may require additional DNA-binding proteins which may be essential for regulating chromatin structure and accessibility in other cell types.

### 2.3. An Interesting Twist in the Plot: The Assembly of the NuRD Complex 

One important unanswered question is how the NuRD subunits are assembled and to which extent the integration of certain paralogs within the complex determines its functions. The observation that NuRD complexes include distinct CHDs during cortical development opens two distinct scenarios regarding the potential mechanisms regulating paralog switch. The first is that, similarly to the BAF45a and BAF45b paralogs of the SWI-SNF chromatin remodelling complex in developing neurons [13], the relative expression level of CHDs primarily determines the composition and the function of the NuRD complex. However, during cortical development, CHD3 and CHD5 are expressed at comparable levels and are integrated into NuRD complexes in a similar manner [14], yet they bind to distinct gene promoters, regulating specific aspects of neural radial migration and differentiation. These findings open several non-mutually exclusive hypotheses, including the different protein–protein or protein–chromatin interactions by specific subunit paralogs. Indeed, the DNA-binding paralogs MBD2 and MBD3 display different affinity for methylated DNA despite both binding to CpG islands [37,38], providing the NuRD complex with the ability to repress or activate genes, depending on the DNA methylation state.

A second mechanism regulating NuRD assembly and chromatin binding may rely on posttranslational modifications (PTMs) of NuRD subunits initiated by extracellular signalling. Although epigenetics, by definition, studies the impact of the external environment on gene expression, how epigenetic enzymes are regulated in response to extracellular stimulation remains a surprisingly understudied area of research [39]. The histone acetylase CREB-binding protein CBP was the first epigenetic factor shown to be phosphorylated in response to neuronal activity, and this event was necessary for the activation of CREB-dependent transcription [40]. Interestingly, the NuRD subunits are among the few epigenetic factors that are known to be targeted by posttranslational modifications. For example, GATAD2A and GATAD2B are sumoylated at distinct sites, and this PTM results in differential affinity for HDAC1 [41]. In cortical neurons, the gaseous messenger nitric oxide (NO) modifies HDAC2 by inducing the nitrosylation of specific cysteines [42,43]. Interestingly, HDAC2 S-nitrosylation principally affects its ability to bind to the promoters of neurotrophin-regulated genes, although alternative mechanisms have been suggested [44]. A mass spectrometry screen aimed at identifying S-nitrosylated nuclear proteins in cortical neurons revealed that most NuRD subunits were modified by NO, including RBBP7 [45]. RBBP7 nitrosylation at a specific cysteine residue regulated the binding to CHD4 and possibly RBBP7 integration within NuRD complexes. Initial evidence obtained in our laboratory indicates that the CHD4 is nitrosylated on two cysteines within the ATPase domain that are essential for its nucleosome sliding activity. In neurons, NO is synthesised by the neuronal NO synthase (nNOS), an enzyme that is activated in response to synaptic activity and calcium signalling [46,47]. Thus, the S-nitrosylation of CHD4 and other NuRD subunits may provide the missing link between extracellular stimuli essential for neuronal development, such as neurotrophins, and the activity of chromatin-modifying enzymes. Given that the composition of chromatin remodelling complexes is known to depend, at least in some cases, on the relative expressions of paralogs [13,14], a scenario can be envisioned by which the combination of subunit expression levels and their PTMs may synergistically regulate the composition of the complexes and specific chromatin interactions. Such complex regulatory mechanisms will ensure that at any stage of neuronal differentiation, specific sets of gene promoters and enhancers are activated, ensuring high transcriptional accuracy. 

### 2.4. When the Good Cop Becomes the Bad Cop: CHDs and Neurodevelopmental Disorders

Mutations of chromatin remodelling complexes have been linked to several neurological disorders ranging from early childhood diseases, such as autism spectrum disorders (ASD) and intellectual disabilities [48], to complex psychiatric syndromes including schizophrenia [49]. 

De novo mutations and/or the deletion of genes encoding NuRD subunits have been involved in many neurodevelopmental disorders (Table 1) [48]. Mutation of the CHD3, usually clustered around the helicase domain, results in a congenital syndrome characterised by craniofacial defects and severe neurodevelopmental delay [50]. CHD4 missense mutations were observed in patients with Sifrim–Hitz–Weiss syndrome (SIHIWES), a disorder associated with global neurodevelopmental delays and cognitive impairment [51,52]. 

More recently, de novo and inherited missense mutations of CHD5 have been found in a small cohort of patients with intellectual disability, epilepsy and behavioural disorder of various severity [53]. Other NuRD subunits have also been found to be altered in other rare neurological disorders. Loss-of-function (LOF) mutations of the GATAD2B gene resulting in RNA nonsense-mediated decay were found in patients with severe motor disabilities and neurodevelopmental delay [54]. Interestingly, pathogenic variants of MBD3 have been found in patients with non-verbal autistic disorder [55,56]. MBD3 belongs to a family of methyl-CpG-binding proteins that includes MeCP2, a gene that is mutated in Rett syndrome, which is an atypical and common autistic disorder mostly found in females [65,66]. Thus, it is possible that MBD3 and MecP2 may share chromatin targets that regulate genes essential for higher cognitive functions. 

In addition to CHD3, CHD4 and CHD5, other highly related members of the CHD superfamily, CHD2, CHD7 and CHD8 have also been implicated in ASD and intellectual disability (Table 1). For example, deletions of CHD2 were detected in patients affected by epilepsy and severe intellectual disability [57,58]. Interestingly, mice lacking CHD2 showed a reduction in GABAergic neurons that may account for the imbalance between excitatory and inhibitory circuits [67]. Mutations of CHD7 are found in patients affected by CHARGE syndrome, a severe clinical condition that affects many organs and is associated with deafness, blindness and often various degrees of intellectual disability [59,60]. Notably, CHD7 interacts with BAF complex subunits in stem cells [61] and with the DNA topoisomerase IIb [68]. Given these unique interactions, loss of CHD7 may interfere with the transcription of specific sets of genes whose expression cannot be rescued by other paralogs. 

Although ASD has been linked to hundreds of risk genes [69,70], CHD8 is widely recognised as one of the most frequently mutated and most penetrant factors implicated in the pathogenesis of the disease [62,63,64]. In rodents, CHD8 regulates brain size, and heterozygous CHD8 mice show different degrees of abnormalities displaying larger brain size and behavioural abnormalities [71,72]. CHD8 also plays an essential role in oligodendrocyte precursor proliferation and differentiation in both the brain and the spinal cord [73]. Interestingly, autistic patients carrying a defective CHD8 gene have shown decreased white matter density and defects of myelination [74]. A recent interesting study has suggested that in human brain organoids, CHD8 shares functional pathways with two ASD high risk genes, SUV420H1 and ARID1B, although they act through distinct molecular targets [75]. Phenotypic analysis revealed that mutation of each gene resulted in defective development of GABAergic neurons and abnormalities of the deep layers of the cortex. Thus, it is conceivable that the cortical inhibitory pathways represent a common target toward which many ASD risk genes converge. 

## 3. Waiting for the Sequel

Chromatin remodelling is increasingly recognised as a primary mechanism that regulates gene expression in neurons. Given the complexity associated with changes in chromatin states, it is not surprising that chromatin remodelling complexes include many subunits that allow them to be extremely flexible in exerting their genome-wide effects on nuclear structure. Yet, precision is equally essential to ensure chromatin remodelling complexes’ assembly on specific promoters and enhancers, depending on the developmental stage and the neuronal cell type. A key future challenge will be to understand how chromatin remodelling complexes such as NuRD are formed, and the signalling that regulates both paralog expression and their assembly on chromatin. Whereas we now know that in some cases the sequential switch of paralog expression dictates their inclusion within the complex [13,14,76], in most cases, paralogs are expressed at similar levels. 

PTMs are a potential key mechanism by which environmental signals may impact NuRD composition by influencing subunit interactions or recruitment to the DNA. Even more intriguingly, PTMs within the ATPase of CHDs or the deacetylase domains of HDACs may directly affect the nucleosome sliding activity and chromatin accessibility to the RNAPolII machinery or other nuclear factors. NO signalling is an especially exciting candidate as it has been shown to modify several NuRD subunits in neurons by means of S-nitrosylation [42,43,45]. Importantly, NO synthesis depends on intracellular calcium signalling, providing a direct link between synaptic activity and chromatin accessibility. In neurons, NuRD interacts with many transcription factors, often in a cell type-specific manner [77]. Determining how these interactions are regulated and whether inclusion of specific paralogs may determine the affinity of NuRD for certain promoters remains an essential question, especially when considering that NuRD has been shown to interact with cortical layer-specific transcription factors [78].

Finally, NuRD paralogs are emerging as high confidence risk factors for a host of neurodevelopmental disorders for which no therapy is available, such as ASDs [79]. Thus, exploring new therapeutical avenues aimed at fine tuning the subunit expression, chromatin remodelling complex composition and the affinity for chromatin may restore some lost neuronal functions. Encouraging results obtained in a model of Rett Syndrome have shown that partially restoring the levels of Mecp2 results in a significant amelioration of the neurological symptoms in mice [80]. Although hundreds of genes have been identified as potential risk factors for complex disorders such as ASD, the discovery that some nuclear factors may share signalling pathways that converge on GABAergic neuronal development [75] raises the exciting possibility that aiming at common molecular targets may provide a novel approach to tackle this complex neurological disorder.

## Figures and Tables

**Table 1:** Summary of NuRD and CHD genes associated with neurodevelopmental disorders.

Gene	Neurodevelopmental Disorders
**NuRD Subunits**	
*CHD3*	Craniofacial defects, developmental delay, language deficits (Snijders Blok–Campeau syndrome) [50]
*CHD4*	Developmental delay, speech and motor delay, cognitive impairment (Sifrim–Hitz–Weiss syndrome) [51,52]
*CHD5*	Language deficits, intellectual disability, epilepsy, behavioural disorder (Parenti–Mignot neurodevelopmental syndrome) [53]
*GATAD2B*	Motor disability, intellectual disability, language deficits, developmental delay, craniofacial abnormalities (GATAD2B-associated neurodevelopmental disorder) [54]
*MBD3*	Non-verbal ASD [55,56]
**Other CHDs**	
*CHD2*	Epilepsy, neurobehavioural disorders, intellectual disability [57,58]
*CHD7*	Intellectual disability, hearing and visual impairments, developmental delay, self-injurious behaviour, sleep problems (CHARGE syndrome) [59,60,61]
*CHD8*	Developmental delay, ASD, behavioural disorder, musculoskeletal defects (Intellectual developmental disorder with autism and macrocephaly, IDDAM) [62,63,64]
